# Computational Screening and Analysis of Lung Cancer Related Non-Synonymous Single Nucleotide Polymorphisms on the Human Kirsten Rat Sarcoma Gene

**DOI:** 10.3390/molecules24101951

**Published:** 2019-05-21

**Authors:** Qiankun Wang, Aamir Mehmood, Heng Wang, Qin Xu, Yi Xiong, Dong-Qing Wei

**Affiliations:** State Key Laboratory of Microbial Metabolism, School of Life Sciences and Biotechnology, Shanghai Jiao Tong University, Shanghai 200240, China; wangqiankun@sjtu.edu.cn (Q.W.); aamirmehmood@sjtu.edu.cn (A.M.); wangheng0802@sjtu.edu.cn (H.W.); xuqin523@sjtu.edu.cn (Q.X.)

**Keywords:** mutation, single nucleotide polymorphism, functional effect, molecular dynamics simulation, structural analysis

## Abstract

The human KRAS (Kirsten rat sarcoma) is an oncogene, involved in the regulation of cell growth and division. The mutations in the KRAS gene have the potential to cause normal cells to become cancerous in human lungs. In the present study, we focus on non-synonymous single nucleotide polymorphisms (nsSNPs), which are point mutations in the DNA sequence leading to the amino acid variants in the encoded protein. To begin with, we developed a pipeline to utilize a set of computational tools in order to obtain the most deleterious nsSNPs (Q22K, Q61P, and Q61R) associated with lung cancer in the human KRAS gene. Furthermore, molecular dynamics simulation and structural analyses of the 3D structures of native and mutant proteins confirmed the impact of these nsSNPs on the stability of the protein. Finally, the experimental results demonstrated that the structural stability of the mutant proteins was worse than that of the native protein. This study provides significant guidance for narrowing down the number of KRAS mutations to be screened as potential diagnostic biomarkers and to better understand the structural and functional mechanisms of the KRAS protein.

## 1. Introduction

Lung cancer remains the most frequent cause of cancer-related death worldwide in the past few decades [1]. Kirsten rat sarcoma (KRAS) viral oncogene homolog mutant tumors constitute the most prevalent targetable molecular subtype of non-small cell lung cancer, which accounts for most of all lung cancer cases [2,3,4]. The KRAS gene encodes a small GTPase membrane-bound protein as the signaling molecule, whose mutations are vital to cellular proliferation and survival. Thus, the precise identification of mutations in the KRAS gene and the encoded protein is extremely important for a clearer understanding of their effects on cancer cell proliferation and survival. However, the experimental methods to detect the functional mutations in a genome or even in a single gene are both time- and resource-consuming. Therefore, it is crucial to develop in silico approaches to identify the functional significant mutations that might aid in the development of cancer cells regarding the KRAS gene.

Single nucleotide polymorphisms (SNPs) are the most frequent type of genetic variations that occur in the coding or non-coding regions of a DNA sequence. There is one variation in every 200–300 bp in the whole human genome. These types of variations account for approximately 90% of the polymorphisms throughout the human genome. Among various types of mutations, the non-synonymous single nucleotide polymorphisms (nsSNPs) which are mutated in the exonic regions will change the protein sequences, affecting the normal gene regulation or natural function of proteins by causing alterations in the transcriptional or translation mechanisms. To date, 12,071 SNPs, including 261 missense mutations, have already been reported in the human KRAS gene deposited in the public database dbSNP [5]. It is vital to efficiently and accurately evaluate the functional effects of SNPs and explore how SNPs affect protein function. In the last decade, a large number of computational tools have been developed to predict the effect of coding non-synonymous variants on a protein’s structure and, ultimately, its function [6,7,8,9,10,11,12]. Since functional sites on proteins are usually shown to be evolutionarily conserved, a web-based tool, ConSurf, has been developed to predict the evolutionary conservation of each amino acid on the protein [13]. The alterations in a protein’s stability upon the incorporation of a mutation also directly affects its function [14,15,16]. Moreover, it is desirable to identify the somatic mutations in the KRAS gene that can result in the development of cancer. On the basis of aims and applications of these computational approaches, the consensus of their prediction outcomes can narrow down the candidate mutations for further validation.

**Figure 1:**
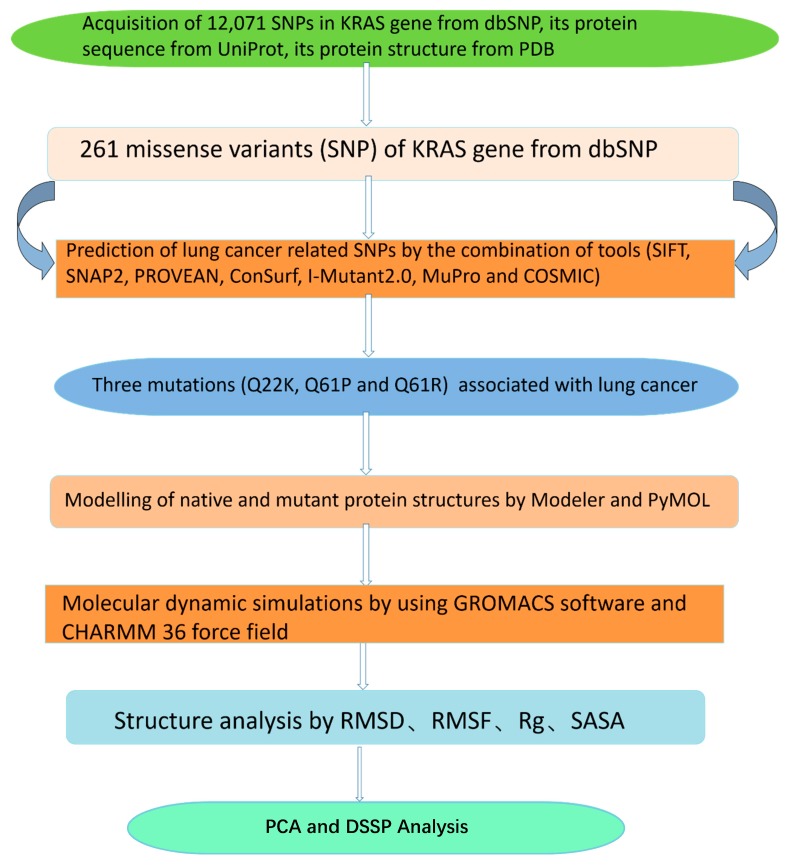
Workflow of our present study.

However, protein functions are not only related to the strictly static structures that are determined by their amino acid sequences, but also highly related to protein dynamics, e.g., the KRAS protein that acts as an on/off switch accompanied by conformational changes in cell signaling. Therefore, we analyzed protein stability via molecular dynamics simulation in order to deeply analyze the structural diversity in mutant KRAS proteins. Inspired by previous studies [17,18], we developed a workflow of computational screening and analysis of lung cancer-related nsSNPs and mutated residues on human KRAS genes and proteins, respectively, which is shown in Figure 1. We believe that our study will help researchers further understand the roles of the KRAS gene and its encoded protein in lung cancer, which will provide guidance for future experimental study.

## 2. Materials and Methods

### 2.1. Data Collection

**Figure 2:**
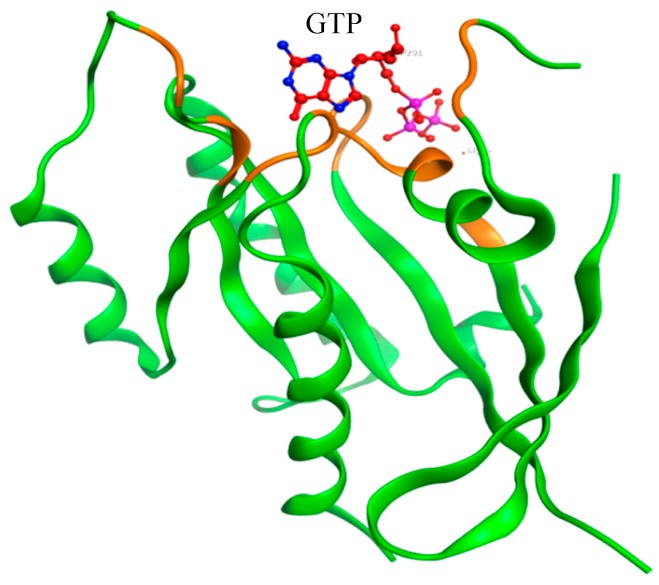
The figure of the Kirsten rat sarcoma (KRAS) protein structure. (The GTP bounding site is shown in orange).

All information about the human KRAS gene was retrieved from public web-based resources. The reported SNP mutations in the KRAS gene was collected from the dbSNP database (http://www.ncbi.nlm.nih.gov/snp/) [5]. The amino acid sequence (UniProt ID: P01116) that encodes a KRAS protein was retrieved from the UniProt database (https://www.uniprot.org/), while the protein 3D crystal structure (shown in Figure 2) was obtained from PDB (Protein Data Bank, http://www.rcsb.org/) with PDB ID 5VQ2 [19,20].

### 2.2. Prediction of Disease Related SNPs

#### 2.2.1. Prediction of Functional Consequences of nsSNPs

The functional effects of nsSNPs were predicted by SIFT (Sorting Intolerant from Tolerant) [7], SNAP2 (screening of non-acceptable polymorphism 2) [10], and PROVEAN (Protein Variation Effect Analyzer) [11]. nsSNPs were assigned as deleterious mutations by the consistent predictions of all three tools.

SIFT (http://sift.bii.a-star.edu.sg) is a program that predicts whether or not an amino acid substitution is responsible for changes in the protein function. Its prediction is based on the physicochemical properties of amino acids in the protein sequence and its sequence homologies [7]. The prediction results of the SIFT program can be categorized into two classes: deleterious and tolerated. The amino acid substitution is predicted to be deleterious if a SIFT score is between 0 and 0.05, while a score between 0.05 and 1 is regarded as tolerable.

SNAP2 (https://rostlab.org/services/snap) is a neural network-based prediction server which identifies the functional effects of amino acid sequence variants [10]. The prediction score ranges from -100 (strongly neutral prediction) to 100 (strong effect prediction), which reflects the likelihood of the single amino acid mutation that may alter the native protein function.

PROVEAN (http://provean.jcvi.org) is a web-based server, which utilizes an alignment-based score approach, for prediction of the functional effect of amino acid variants [11]. We submitted the query protein sequence and amino acid variations to the PROVEAN server, which performed a BLAST search to collect homologous sequences, and the scores for each mutation were calculated. The threshold for PROVEAN scores was set to −2.5 to discriminate deleterious substitutions from neutral ones.

#### 2.2.2. Estimation of Evolutionary Conservation of nsSNPs

The level of evolutionary conservation of each sequence position corresponds to the evolutionary rate, which is not constant among all amino acids in a protein. The amino acid positions which evolve slowly are commonly considered as conserved sites that are important for protein structure and function. The ConSurf server (http://consurf.tau.ac.il/) was used to estimate the level of evolutionary conservation of amino acid positions in a protein, based on the phylogenetic relationships between homologous sequences [13,21,22]. We submitted both the protein sequence and structure to the ConSurf server, which calculates the conservation scores partitioned into a discrete scale of nine bins. The positions with bin 9 indicate the most conserved sites, while the positions with bin 1 indicate the most variable sites.

#### 2.2.3. Prediction of Protein Change Stability of nsSNPs

Accurate prediction of protein stability changes upon single point mutations is important for understanding protein structure and function. In the present study, we used MuPro [14] and I-Mutant 2.0 [15] to predict protein stability changes for the SNPs. MuPro (http://mupro.proteomics.ics.uci.edu/) is a support vector machine-based tool to predict protein stability changes for single amino acid mutations based on protein sequence or/and structural features [14]. I-Mutant 2.0 (http://folding.biofold.org/i-mutant/i-mutant2.0.html) is another support vector machine-based web tool to make automatic predictions of protein stability changes upon single point mutations [15]. We uploaded the protein sequence, position of mutation, and the mutant residue, and the protein stability was predicted at default temperatures and pH. The reliability index value of the prediction that ranges from 0 (unreliable) to 10 (reliable) was also calculated.

#### 2.2.4. Identification of Somatic Mutations that can Cause Cancer

Furthermore, we identify the somatic mutations that can cause cancer in the KRAS gene. The COSMIC (Catalogue of Somatic Mutations in Cancer, https://cancer.sanger.ac.uk/cosmic/) website was developed for curating the somatic mutations information related to human cancer [23].

### 2.3. Modeling of Native and Mutant KRAS Proteins

**Figure 3:**
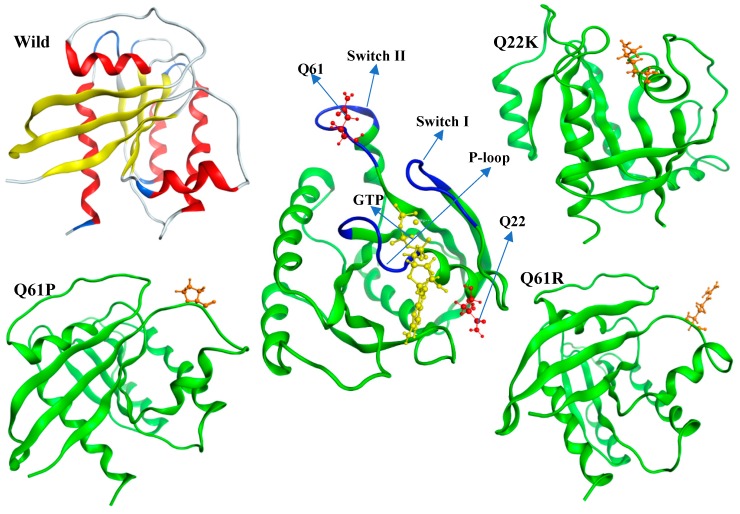
Graphical representations of selected single nucleotide polymorphisms (SNPs) and the GTP binding site in the protein structure.

The crystal structure of the KRAS protein was obtained from PDB (Entry ID: 5VQ2; Chain: A; Resolution: 1.96 Å) [20]. All water molecules and ligands were removed from the crystal structure, and the Modeler 9.19 package was used to map the missing parts of structure on the wild-type (WT) protein [24]. Moreover, the WT structure was mutated by each one of the three most deleterious mutants predicted in the previous sections. The three structures of mutant (MT) proteins, such as Q22K, Q61R, and Q61P, were modeled by making a point mutation in the wild-type (WT) protein structure using PyMOL software. Then, we used the DynaMut [25] web server (http://biosig.unimelb.edu.au/dynamut) for an initial assessment of the impact of point mutations on protein dynamics and stability. The WT and three MT structures are shown in Figure 3.

### 2.4. Molecular Dynamics Simulation and Trajectories Analysis

We used molecular dynamics simulation (MD) techniques to investigate the mechanism of structural impacts of the mutations on KRAS. MDs were performed using GROMACS 5.1.2 [26,27] software on an Ubuntu 16.04.5 operating system running on a machine equipped with a 12 terabyte hard-disk, 63 gigabytes RAM, Intel(R) Xeon(R) CPU E5-2640 processor. The Modeler 9.19 package was used to refine the structure of the WT protein, and the PyMOL software was used to map the mutations on the structures of mutant proteins. Then, the protein systems were solvated in a cubic box with SPC (simple point charge) water molecules and the walls were located ≥12 Å from all protein atoms. The box size was set to 4.256 nm × 4.061 nm × 4.142 nm, with box vectors of 6.7 × 6.7 × 6.7 nm, and box angles were kept at 90° for each side. The total number of atoms in WT, Q22K, Q61P, and Q61R were 29,063, 30,817, 29,638, and 30,554, respectively. The simulation was performed using the CHARMM 36 force field [28] at a neutral pH, which was neutralized by adding a number of Na+ counter ions (7, 6, 7, and 6 for WT, Q22K, Q61P, and Q61R, respectively). The energy of each solvated system was minimized with 50,000 iterations, and the steepest descent minimization was terminated when the maximum force was below 1000 KJ/mol^−1^/nm^−1^. After the process of energy minimization, the system was equilibrated with pressure (1 bar) and constant temperature (310 K) at a time step of 2 fs. The LINCS (LINear Constraint SolVer) [29] constraints and non-bonded pair list were updated every 10 steps under the position restraint conditions for the heavy atoms. Electrostatic interactions were calculated using the particle mesh Ewald method. The v-rescale (modified Berendsen thermostat) temperature coupling method [30] was used to maintain the constant temperature inside the box. Finally, all the systems were simulated for a duration of 100 ns MD simulations and the coordinates were saved after an interval of every 2 ps.

After the completion of MD, trajectories were analyzed to compare and observe the structural deviation among the KRAS wild-type and mutant structures (MT). The root mean square deviation (RMSD), root mean square fluctuation (RMSF), radius of gyration (Rg), solvent-accessible surface area (SASA), and secondary structure calculation, were calculated by using the g_rms, g_rmsf, g_gyrate, g_sasa, and do dssp utilities of Gromacs.

### 2.5. Principal Component Analysis

Principal components analysis (PCA) or essential dynamics (ED) were used to reduce the dimensionality of the molecular dynamics simulations data in order to identify the configuration space of anharmonic motion with only a few degrees of freedom. PCA is a method that analyzes the MD trajectory and extracts dominant modes in the overall molecular motion. The motion of structures in a multidimensional space was identified by the most vital eigenvectors projection in Cartesian trajectory coordinates. In the ED analysis, we constructed a covariance matrix of WT and MTs backbone Cα atoms simulation trajectories which removed the rotation and translational movements. Furthermore, we calculated the eigenvectors and eigenvalues of the covariance matrices, and the projection of the first two principal components. We achieved the principal component analysis of trajectories using the Gromacs built-in utilities, such as gmx covar and gmx anaeig.

## 3. Results and Discussion

### 3.1. SNP Data Set from dbSNP

**Figure 4:**
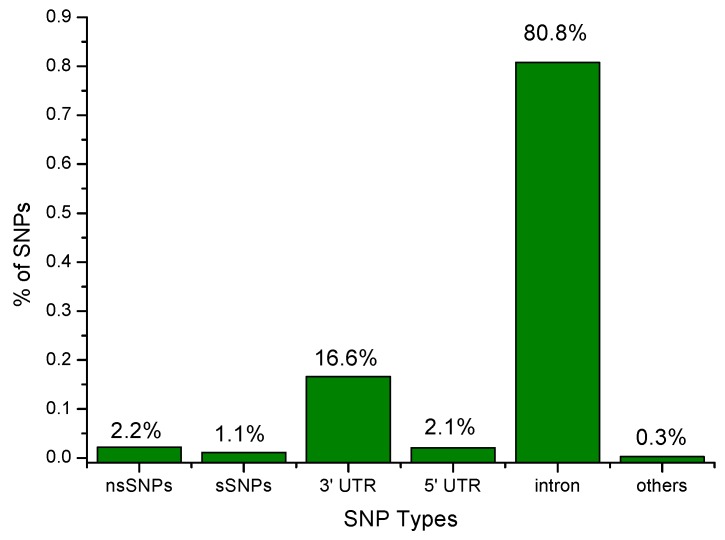
Distribution of different types of SNPs in the KRAS gene.

The dbSNP database contains a total of 12,071 SNPs for the KRAS gene. Among the 12,071 SNPs, 261 (2.2%) are missense mutations, which is a type of nonsynonymous substitution in DNA sequences, 131 (1.1%) are coding synonymous SNPs, 2005 (16.6%) SNPs are in the mRNA 3′UTR region, 257 (2.1%) are in the 5′UTR region, and 9754 (80.8%) are in the intronic region. The remaining 42 (0.3%) are nonsense, frame shift, 3′ splice site, 5′ splice site and stop gained SNPs. The distribution of SNPs is illustrated in Figure 4. We selected the 261 missense mutations for further investigation on the basis of our proposed workflow. Out of 261 mutations in the KRAS gene, 106 of them were mapped to the amino acid positions on the protein sequence (UniProt ID: P01116). Next to this, we identified the most likely pathogenic mutations that confer susceptibility to human diseases regarding the KRAS gene, with six in silico tools—SIFT, SNAP2, PROVEAN, ConSurf, MuPro, and I-Mutant2.0. In order to improve the prediction accuracy, we combined those computational methods that are based on the protein structural and/or functional parameters with necessary evolutionary information. Finally, we used the COSMIC database to identify the three nsSNPs associated with lung cancer.

#### 3.1.1. Screening of Missense SNPs Based on Functional Analysis

A total of 106 missense mutations were used for the prediction of their functional effects via SIFT, SNAP2, and PROVEAN tools. Out of 106 nsSNPs, SIFT predicted 70 nsSNPs as ‘intolerant’, with scores ≤ 0.05 and the remaining 36 nsSNPs were predicted as ‘tolerated’, with a score greater than 0.05. SNAP2 predicted 90 nsSNPs as ‘effect’, with scores > 0, out of which 54 were predicted as ‘effect’ with scores ranging from 50 to 100, and 36 nsSNPs were predicted as ‘effect’ with scores ranging from 0 to 50. The remaining 16 nsSNPs were predicted as ‘neutral’, with scores < 0. Moreover, all the missense SNPs were also analyzed by PROVEAN. The mutations with scores less than or equal to −2.5, in case of PROVEAN, were considered ‘deleterious’, while the mutations with scores greater than −2.5 were predicted to be ‘neutral’. According to the default threshold, out of 106 nsSNPs, 78 were predicted to be ‘deleterious’ and 28 nsSNPs were predicted to be ‘neutral’. The predicted results of all three tools are shown in Table 1. The 64 nsSNPs (shown in bold) were predicted to be deleterious and were chosen for further investigation.

#### 3.1.2. Analysis of Deleterious nsSNPs Based on the Residue Evolutionary Conservation

**Figure 5:**
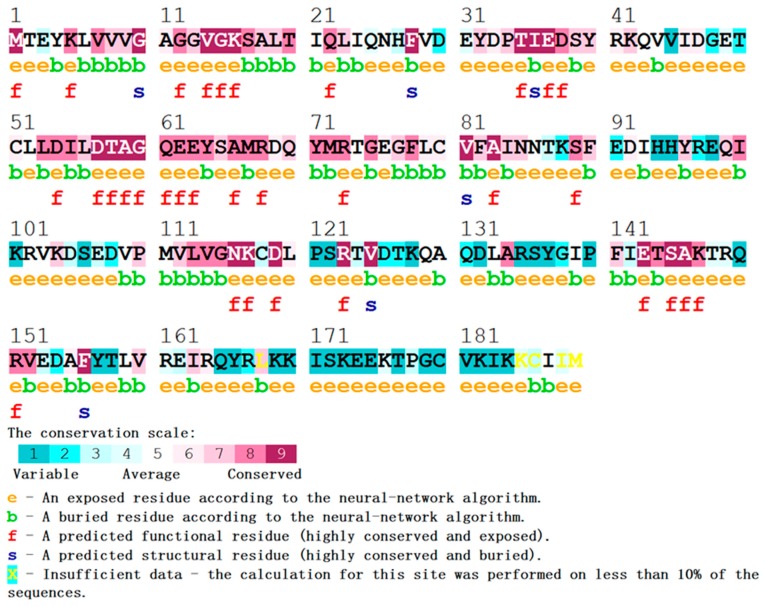
ConSurf output using the UniRef90 protein database. Colors of the ConSurf output indicate the degree of sequence conservation. The two pole colors (blue, purple) indicate variability and conservation, respectively. Residues are predicted to be exposed (e), buried (b), functional (i.e., highly conserved and exposed; f), structural (i.e., highly conserved and buried; s), or have insufficient data (x). Numbers indicate the residue number of KRAS protein.

The conservational level of an amino acid highly affects the protein’s overall structure and function. Evolutionary information of proteins is vital for understanding those variations which are disease-causing mutations. Therefore, another round of confirmation was carried out to test the validity of our selected mutations, by analyzing the degree of conservation for a particular amino acid via the ConSurf server. ConSurf is an evolutionary conservation analysis tool that constructs a protein structural representation map with the colorimetric conservation score. The conservation scale in the range of 7 to 9 is considered to be conserved, while those in the range of 4 to 6 and 1 to 3 are considered to be average and variable, respectively. As we all know, disease-causing mutations often exist in the functional domains and reside on highly conserved positions. Based on the protein structural representation map (shown in Figure 5) and the results mentioned in Table 2, 32 mutations (shown in Table 2 with bold) out of 64 nsSNPs were observed to be highly conserved and were found to be located on the highly exposed accessible surfaces. All of the 32 mutations were further subjected to stability inspection.

#### 3.1.3. Screening of Deleterious nsSNPs Based on the Stability Analysis

In this step, the stability analysis of our 32 nsSNPs was conducted with I-Mutant 2.0 [15] and MuPro [14]. The MuPro server predicted 31 nsSNPs to be ‘decrease’ and the remaining 1 to bes ‘increase’. A negative score obtained from MuPro means the mutation decreases the protein’s structure stability. On the contrary, if the score is >0, it means the mutation increases the protein’s structure stability. At a pH of 7.0 and a temperature of 25 °C, the I-Mutant 2.0 was used to evaluate the stability of the mutants, whether they will cause a change in the protein structure stability. For this purpose, the free energy value (DDG value) and reliability index (RI) were computed. According to I-Mutant’s threshold, a DDG score less than 0 (<0) or greater than 0 (>0) will be claimed as decreased or increased stability, respectively. I-Mutant 2.0 showed that 28 nsSNPs (shown in Table 3 with bold) have decreased the stability of the protein structure and 4 nsSNPs have increased the stability of the protein structure.

#### 3.1.4. Lung Cancer Related Mutations by COSMIC Database

By combining the predictions of the SIFT, SNAP2, PROVEAN, Consurf, I-Mutant, and MuPro tools, of 106 nsSNPs, only 28 nsSNPs were found to be more deleterious. Furthermore, we used the COSMIC database to confirm the nsSNPs which confer a lung cancer phenotype. From the COSMIC database, we identified that rs121913236 (Q22K) and rs121913240 (Q61P and Q61R) cause lung tumors. Hence, those three nsSNPs structures were selected for further MD analysis.

### 3.2. Molecular Dynamics Simulation

Mutations may cause conformational changes in protein structures, which might lead to zero or poor protein function or production. In order to better understand the functional and structural behaviors of the prioritized deleterious mutations, we used molecular dynamics simulation to analyze the native and mutant (Q22K, Q61P, and Q61R) proteins. Four systems were built and 100 ns MDs were run by Gromacs. The trajectory files were generated after the molecular dynamics simulation, and various analyses, such as root mean square deviation (RMSD), root mean square fluctuation (RMSF), radius of gyration (Rg), solvent-accessible surface area (SASA) variations, and PCA analysis for the native and the three mutant structures were carried out.

#### 3.2.1. Structural Stability Analysis

**Figure 6:**
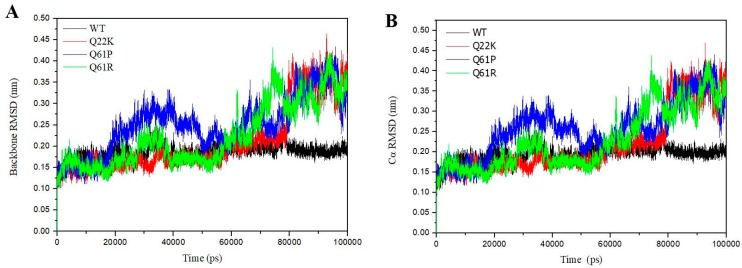
Backbone RMSD (**A**) and Cα-RMSD (**B**) for the wild (black) and Q22K (red), Q61P (blue), and Q61R (green).

The RMSD value of backbone and C_α_ atoms in WT and three MTs were calculated to assess the conformational stability of the protein during the simulations. As seen in Figure 6A (backbone-RMSD), the mutations caused a notable difference in RMSD pattern between WT and MTs. The RMSD values of the three mutants were constantly higher fluctuated and increased to 80 ns, but after that the three mutants’ RMSD values were stable, with a comparatively lower fluctuation rate of around 0.35 nm. For the native protein, there was just one sharp increase in the first 9 ns and two lower fluctuations at 28–40 ns and 75–80 ns. The average backbone-RMSD values for WT and the three MTs are 0.1853, 0.2108, 0.2504, and 0.2240 nm, respectively (shown in Table 4). We compared the average backbone-RMSD values, which showed the order Q61P > Q61R > Q22K > WT. Meanwhile, the Figure 6B (Cα-RMSD) graph is similar to Figure 6A (backbone-RMSD), and shows the rank of collected RMSD values: Q61P (0.2562) > Q61R (0.2294) > Q22K (0.2173) > WT (0.1933) (Table 4). There is an interesting result; it showed a higher average value for mutants and a lower average value for the native protein. From the results, we reached the conclusion that MTs could change the protein structure and mutations are not stable like native proteins. Since the protein needs a proper and stable structure to perform its function, we can speculate that the mutations alter the stability and activity of the protein.

#### 3.2.2. Structural Flexibility Analysis

**Figure 7:**
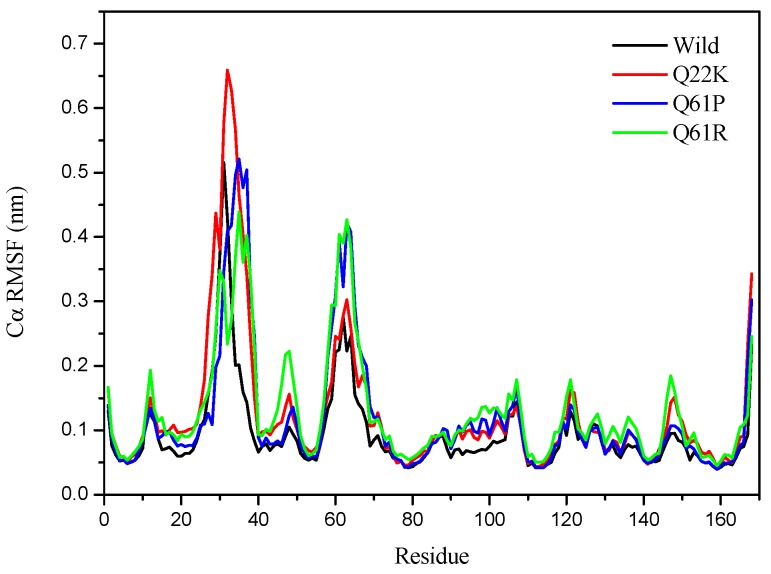
Cα RMSF for the Wild (black), Q22K (red), Q61P (blue) and Q61R (green).

The RMSF values of WT and the three MTs Cα amino acids were calculated to determine whether the mutants affected the dynamic behavior of the residue. As seen in Figure 7, different fluctuating behaviors were observed in mutants and WT. All cases of mutant simulations had higher average Cα-RMSF values than the WT simulation, with the average RMSF values for mutants being 0.1251, 0.1183, 0.1289 nm for Q22K, Q61P, and Q61R, respectively, while the RMSF value for WT is 0.0939 nm (Table 4). According to the fluctuation score, we ranked the collected values as follows: Q61R > Q22K > Q61P > Wild. These results show that a higher degree of flexibility was observed in mutants (Q22K, Q61P, and Q61R) than that of the native protein structure. For a small protein, a fluctuation value below 2 Å is acceptable. Figure 7 shows that, for all cases of WT and MTs, most of the higher fluctuation occurred in the N-terminal domain and residues from Ala11 to Thr74 showed significant fluctuation. In the C-terminus domain, they also showed higher fluctuations in all cases of WT and the three mutations. At the same time, Figure 7 shows that the RMSF value of each mutant residue was greater than that of WT. Therefore, the results indicate that mutation affected the flexibility of the whole protein, not just of the residual level. Overall, the mutations alter the flexibility and activity of proteins in our cases. The RMSF results are in agreement with that of the RMSD.

#### 3.2.3. Structural Compactness Analysis

**Figure 8:**
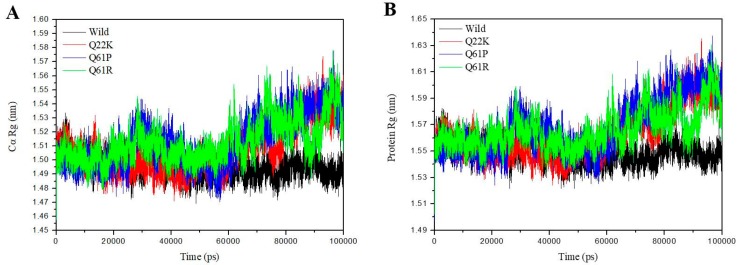
Radius of gyration of (**A**) Ca atoms and (**B**) Proteins of the Wild (black), Q22K (red), Q61P (blue), and Q61R (green).

The radius of gyration (Rg) is defined as the mass-weight root mean square distance of a collection of atoms from their common center of mass. Rg provides an insight into the overall dimension of the protein. Hence, Rg is also a vital parameter to describe the dynamic stability and compactness of the total protein systems. The Rg was plotted for both Cα atoms and proteins against time over the whole 100 ns simulations at 310 K. From Figure 8A (Cα–Rg), the mutant (Q22K, Q61P, and Q61R) structures show a notable fluctuation and a clearly higher average Rg value than that of the native structure. The three mutant curves are similar to the native in the 0–60 ns time period, but after that native protein showed a stable Rg value, while the three mutant curves showed a sharp increase and higher fluctuation during the last simulation time. From Figure 8B (protein–Rg), it can be seen that the trend is similar to Figure 8A (Cα–Rg). Based on the Rg graph, it was found that the conformations of these three mutants are getting more dispersed and becoming significantly different to the native conformation in the simulation time period, whereas the native structure was stable compared to the mutant. The average Cα–Rg values were 1.4960, 1.5072, 1.5130, and 1.5128 nm in native and mutant (Q22K, Q61P, and Q61R) structures, respectively (shown in Table 4). According to the fluctuation scores, we ranked the collected values as follows: Q61P > Q61R > Q22K > Wild. The Rg results suggest that the mutation changed the protein structure with increasing flexibility in its conformation. In all, the Rg results are in good agreement with that of RMSD and RMSF.

#### 3.2.4. SASA Analysis

**Figure 9:**
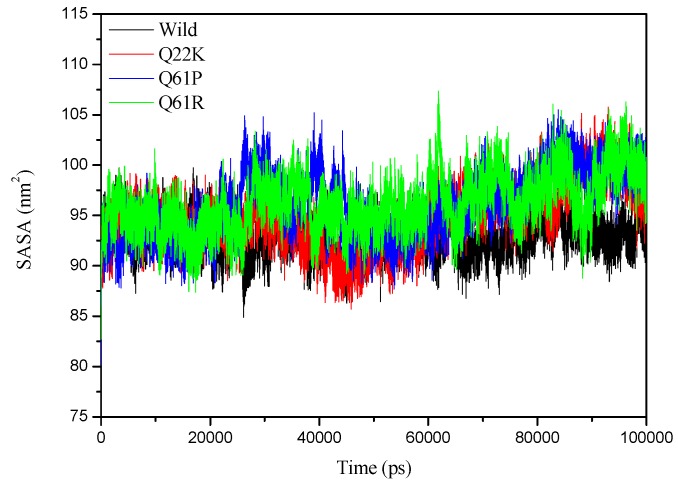
Solvent-accessible surface area (SASA) of proteins of the Wild (black), Q22K (red), Q61P (blue), and Q61R (green).

The solvent-accessible surface area (SASA) is the surface area of a biomolecule that is accessible to a solvent. It is used to measure the degree to which an amino acid is exposed to its environments. A lower SASA value indicates a compact protein structure, while a higher SASA value indicates a diffused structure. An increase or decrease in SASA value indicates a change in the protein’s structural conformation. The SASA values of the WT and three MT proteins were analyzed for predicting how the mutations affect the structure of the native protein. The SASA values calculated for the WT and three MTs with time are shown in Figure 9, and average SASA values are depicted in Table 4. The figure clearly shows that the three mutant proteins have a higher SASA value than that the native protein. Results from Table 4 clearly indicate that the average SASA value of the WT protein is smaller than that of the MT proteins. The rank of collected average SASA values are listed as: Q61R (96.159 nm^2^) > Q61P (96.109 nm^2^) > Q22K (94.806 nm^2^) > WT (93.008 nm^2^) (Table 4). These values represent that mutants may change the protein’s tertiary structure. The three mutant structures increased the values of SASA so that the structure expands in comparison to the native structure. Therefore, the SASA results are also in agreement with the RMSD, RMSF, and Rg results.

#### 3.2.5. Principal Component Analysis

**Figure 10:**
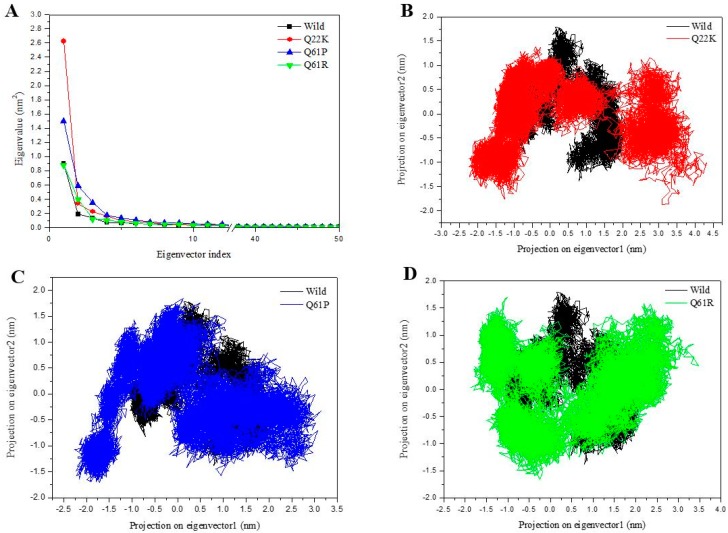
Principal component analysis. (**A**) Eigenvalue for the first fifty modes of motion of Wild (black), Q22K (red), Q61P(blue), and Q61R (green), projection of the motion for native and mutant in phase space along the PC1 and PC2 for Wild (black), Q22K (**B**, red), Q61P(**C**, blue), and Q61R (**D**, green).

Principal component analysis (PCA) or essential dynamics (ED) analysis are widely used for predicting the dynamic behaviors of a protein. We performed PCA to identify large-scale collective motions of the WT and three MTs on the trajectories generated by our simulations. The eigenvalues of the WT and MT proteins were plotted against the corresponding eigenvector index for the first fifty modes of motion (Figure 10A). The eigenvalues indicated fluctuations of the eigenvector in the hyperspace, and the figure shows that only a few eigenvectors have larger eigenvalues which played a major role in the overall motion of the WT and MTs. It was found that the first five eigenvectors had significantly dominant motions with a higher eigenvalue, and the remaining eigenvectors were observed to have extremely low eigenvalues in the overall system (Figure 10A). Thus, we calculated the percentage of first five principal components occupying up to the total observed fifty modes of motion. Throughout the four systems, the first five eigenvectors accounted for 67.32%, 79.94%, 73.87%, and 72.02% of the WT and three MTs (Q22K, Q61P, and Q61R) respectively. These analyses suggest that the mutation changed the structural dynamics of the mutant proteins.

Furthermore, we selected the first two principal components (PC1, PC2) to analyze their projection of trajectories during the WT and MT simulations in the phase space (shown in Figure 10B–D). During the four system simulations, the results clearly show that the WT protein covered a smaller region of phase space, while all three MTs occupied a larger region of phase space. Therefore, the PCA results suggest that the WT protein is more stable than the three MT proteins, and these mutations highly altered the structural stability and flexibility. In short, the PCA results are also in agreement with the RMSD, RMSF, Rg, and SASA results, which enhances the validity of the performed analysis.

#### 3.2.6. Secondary Structure Analysis

**Figure 11:**
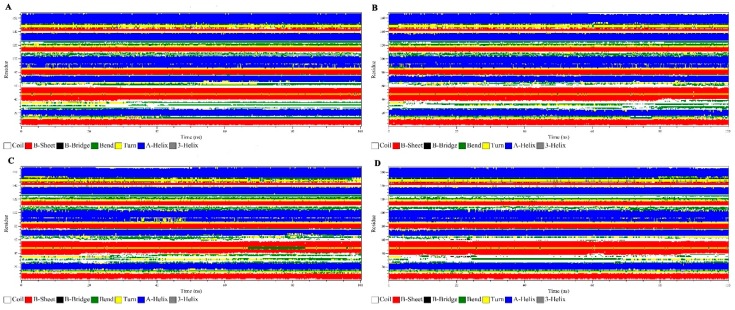
Time evolution of the secondary structural elements of the wild and mutant (Q22K, Q61P, and Q61R) KRAS proteins at 310 K (DSSP classification). (**A**) Wild, (**B**) mutant Q22K, (**C**) mutant Q61P, (**D**) mutant Q61R.

**Figure 12:**
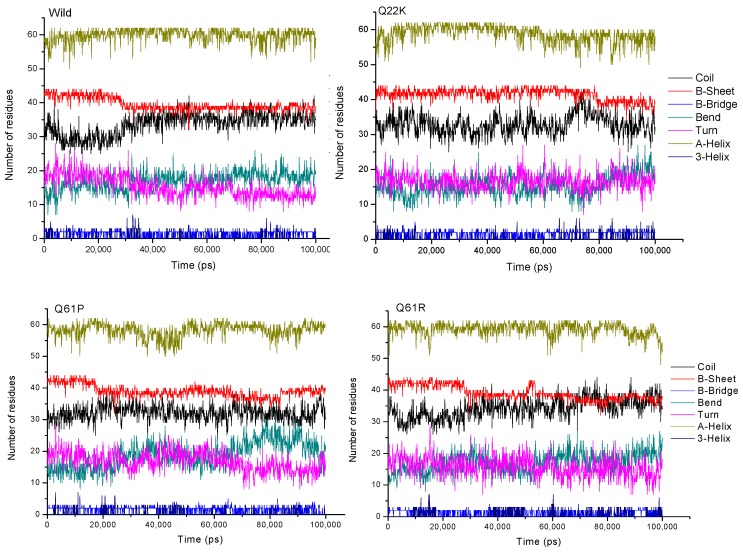
Number of residues involved in the formation of each type of secondary structure for wild and mutant (Q22K, Q61P, and Q61R) KRAS proteins, with respect to simulation time.

The secondary structure of the protein was investigated for general alterations in the domain layout. In order to investigate the secondary structure changes over time, the built-in do_dssp function of GROMACS was used. The secondary structure of the four protein systems (WT, Q22K, Q61P, and Q61R) during the 100 ns MD simulation was retrieved with a follow up step every 100 ps. Figure 11 represents the four secondary structures’ layout, illustrating that non-significant changes can be observed in the case of mutant layouts as compared to the wild-type. Hence, the number of residues involved in the formation of each type of secondary structure for all the examined protein systems was particularly monitored to address the protein structure and outcomes more clearly, which are presented in the Figure 12. From Figure 12, the changes in the given protein secondary structure pattern illustration are not very clear. Therefore, it was necessary to obtain more secondary structure information to validate our obtained outcomes. The total secondary structure content averaged over the trajectories for four protein systems is given in Table 5. The listed values in the given table indicate the percentages of secondary structures, which reveals a minor difference between the WT and MT systems. This confirmed the results shown in the secondary structure diagram.

**Figure 13:**
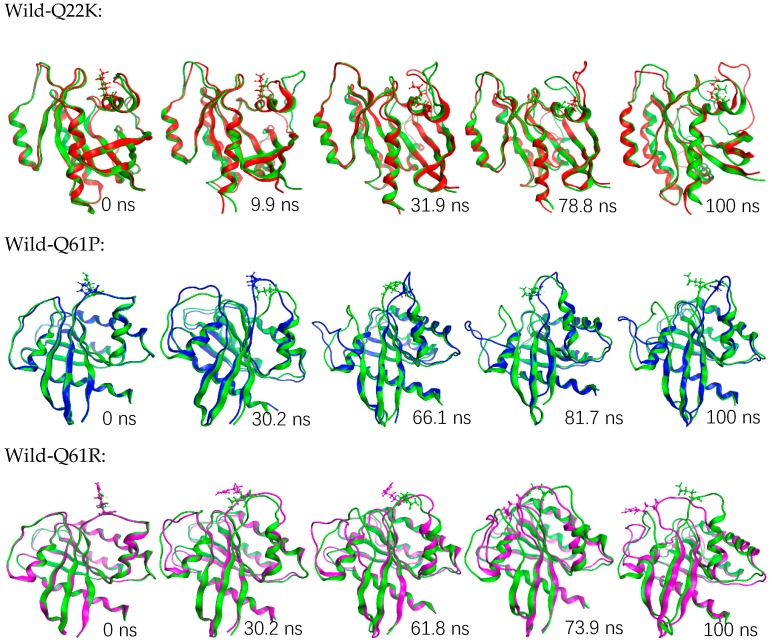
Snapshots of wild and mutant (Q22K, Q61P and Q61R) KRAS protein conformation at different simulation time steps.

In the previous analysis, we learned that mutation changed the stability and flexibility of the protein. The mutant will cause the protein stability to weaken and the flexibility to increase. However, in the secondary structure analysis, we found that the mutation did not cause the protein to produce an obvious conformational drift. To examine how the structure changed and affected the functions upon the incorporation of these mutations, we analyzed the wild and mutants (Q22K, Q61P, and Q61R) superimposed structures at various time steps, depending on backbone RMSD (shown in Figure 13). The results show that mutants can cause severe turbulences in several loop regions of the protein. For example, position 22 is inside the protein active pocket and the Q22K will lead to significant changes in the loop region (HIS27-GLU37, HIS-PHE-VAL-ASP-GLU-TYR-ASP- PRO-THR-ILE-GLU) which constitutes the protein’s active pocket, and position 61 lies in switch II (amino acids 60-67) which regulates ligand binding to the KRAS protein. The mutants Q61P and Q61R also cause significant changes in the loop regions (THR58-SER65, THR-ALA-GLY-GLN-GLU-GLU- TYR-SER) and (HIS27-GLU37, HIS-PHE-VAL-ASP-GLU-TYR-ASP-PRO-THR-ILE-GLU). In addition, we also extracted the average structure (Wild, Q22K, Q61P, and Q61R) from the trajectory to analyze the interaction structure plot (Figure S1) with LIGPLOT, and analyzed the hydrogen bonds (Figure S2) between the mutation and the direct neighborhood during the simulation. Both results showed that these three mutants led to a considerable decrease in the number of H-bonds for the mutants and their direct neighborhood. These results indicate that point mutation can directly affect the stability of its interaction with the surrounding residues, which in turn results in the change in protein structure.

The results further indicated that mutant (Q22K, Q61P, Q61R) structures has more f instability and flexibility than the wild structure. It is well known that the KRAS protein is a signal switch molecule that regulates cell fates by coupling receptor activation to downstream effector pathways that control diverse cellular responses, including proliferation, differentiation, and survival. The three mutations we chose can change the stability of the natural KRAS protein; these changes induce protein structural alterations, which in turn affect its function, as reported by Chikan et al. [31]. Therefore, these three mutations may cause the KRAS protein to be unable to perform its native function. That is to say, its binding mode with GTP and GDP cannot be normally converted, which makes the regulation of the KRAS protein on the downstream invalid, leading to various diseases. Much research on KRAS mutations mainly focuses on targeted therapy drugs [32,33,34], testing for KRAS mutations [35], and clinical research [36,37]. We used molecular dynamics to study the conformation of mutated proteins, which is of great significance to the follow-up study of molecular mechanism after mutation and drug design.

## 4. Conclusions

In this study, we combined computational screening approaches and a public pathogenic database to identify three disease-associated nsSNPs (Q22k, Q61P, and Q61R), which are confirmed to be highly deleterious and can play a crucial role in the progression of lung cancer. Furthermore, the molecular dynamics simulation approach was used to validate the effect of these deleterious point mutations on the KRAS protein structure. The stability, flexibility, and compactness alterations in the mutants were observed in the RMSD, RMSF, and Rg graphs. The experimental results were further supported by an increase in the SASA values and a larger region of phase space in the PCA analysis. Finally, the secondary structure analysis results also suggest that WTs have a more stable cluster in comparison to MTs, and mutation induces structural change in WT proteins. All the results proved that these three mutations can alter the stability and function of the native KRAS protein. Overall, our study provides a comprehensive pipeline to detect the lung cancer-associated nsSNPs which are highly responsible for affecting the native protein dynamics that make the carrier, i.e., humans, more susceptible to developing oncogenic conditions. This study also provides insight and guidance for the design of therapeutic strategies against human lung cancer in the future. It should be noted that PyMol manipulation possibly produces destabilization in the presented study. The manipulation of the structure can introduce strain that cannot really be removed later by energy minimization and relaxation. We will devote ourselves to the resolution of this potential problem in further studies.

## Figures and Tables

**Table 1:** The functional consequences of missense SNPs predicted by Sorting Intolerant from Tolerant (SIFT), screening of non-acceptable polymorphism 2 (SNAP2), and Protein Variation Effect Analyzer (PROVEAN). SNPs indicated in bold are predicted to be highly deleterious and are selected for further evaluation.

rs ID	Variant	SIFT		SNAP2		PROVEAN	
		Prediction	Score	Prediction	Score	Prediction	Score
**rs17851045**	**Q61H**	**intolerant**	**0**	**effect**	**94**	**Deleterious**	**−4.556**
rs104886028	M72I	tolerated	0.25	effect	7	Deleterious	−3.376
**rs104886029**	**A59V**	**intolerant**	**0**	**effect**	**38**	**Deleterious**	**−3.839**
**rs104894359**	**G60S**	**intolerant**	**0**	**effect**	**73**	**Deleterious**	**−5.817**
**rs104894359**	**G60R**	**intolerant**	**0**	**effect**	**99**	**Deleterious**	**−7.758**
**rs104894361**	**K5N**	**intolerant**	**0**	**effect**	**90**	**Deleterious**	**−3.836**
**rs104894362**	**F156L**	**intolerant**	**0**	**effect**	**65**	**Deleterious**	**−5.310**
**rs104894364**	**T58I**	**intolerant**	**0**	**effect**	**97**	**Deleterious**	**−5.823**
**rs104894365**	**V14I**	**intolerant**	**0**	**effect**	**96**	**Neutral**	**−0.819**
**rs104894366**	**P34R**	**intolerant**	**0**	**effect**	**96**	**Deleterious**	**−7.598**
**rs104894367**	**V152G**	**intolerant**	**0**	**effect**	**75**	**Deleterious**	**−5.873**
**rs112445441**	**G13D**	**intolerant**	**0**	**effect**	**98**	**Deleterious**	**−5.403**
**rs121913236**	**Q22K**	**intolerant**	**0**	**effect**	**55**	**Deleterious**	**−3.313**
**rs121913238**	**Q61K**	**intolerant**	**0.01**	**effect**	**69**	**Deleterious**	**−3.588**
**rs121913239**	**Q61E**	**intolerant**	**0.01**	**effect**	**58**	**Deleterious**	**−2.772**
**rs121913240**	**Q61P**	**intolerant**	**0.01**	**effect**	**63**	**Deleterious**	**−5.602**
**rs121913241**	**Q61R**	**intolerant**	**0.01**	**effect**	**63**	**Deleterious**	**−3.455**
**rs121913242**	**Q61L**	**intolerant**	**0.01**	**effect**	**93**	**Deleterious**	**−6.507**
**rs121913527**	**A146P**	**intolerant**	**0**	**effect**	**82**	**Deleterious**	**−4.513**
**rs121913528**	**A59T**	**intolerant**	**0.01**	**effect**	**40**	**Deleterious**	**−3.727**
**rs121913528**	**A59S**	**intolerant**	**0**	**effect**	**35**	**Deleterious**	**−2.701**
**rs121913529**	**G12D**	**intolerant**	**0**	**effect**	**99**	**Deleterious**	**−5.373**
**rs121913531**	**G12A**	**intolerant**	**0**	**effect**	**96**	**Deleterious**	**−4.621**
**rs121913534**	**G12V**	**intolerant**	**0**	**effect**	**98**	**Deleterious**	**−7.113**
**rs121913530**	**G12S**	**intolerant**	**0**	**effect**	**97**	**Deleterious**	**−4.443**
**rs121913532**	**G12R**	**intolerant**	**0.04**	**effect**	**99**	**Deleterious**	**−6.183**
**rs121913533**	**G12C**	**intolerant**	**0**	**effect**	**94**	**Deleterious**	**−7.161**
**rs121913535**	**G13C**	**intolerant**	**0**	**effect**	**92**	**Deleterious**	**−7.619**
**rs121913538**	**L19F**	**intolerant**	**0**	**effect**	**54**	**Deleterious**	**−3.373**
**rs193929331**	**K5E**	**intolerant**	**0**	**effect**	**83**	**Deleterious**	**−3.154**
rs200970347	G179S	tolerated	0.06	neutral	-36	Neutral	−1.374
rs201170656	M189L	tolerated	1	neutral	-67	Neutral	−0.162
**rs202247812**	**N116S**	**intolerant**	**0**	**effect**	**81**	**Deleterious**	**−4.564**
**rs372793780**	**R164Q**	**intolerant**	**0.02**	**effect**	**53**	**Deleterious**	**−2.610**
**rs387907205**	**Y71H**	**intolerant**	**0**	**effect**	**75**	**Deleterious**	**−4.454**
**rs387907205**	**Y71D**	**intolerant**	**0**	**effect**	**86**	**Deleterious**	**−9.374**
**rs387907206**	**K147E**	**intolerant**	**0**	**effect**	**83**	**Deleterious**	**−3.624**
**rs397517041**	**V152F**	**intolerant**	**0**	**effect**	**65**	**Deleterious**	**−4.424**
**rs397517042**	**F156I**	**intolerant**	**0**	**effect**	**62**	**Deleterious**	**−5.312**
**rs397517042**	**F156V**	**intolerant**	**0**	**effect**	**64**	**Deleterious**	**−6.196**
rs397517476	Y166N	tolerated	0.28	effect	18	Deleterious	−3.387
rs397517476	Y166H	tolerated	0.49	effect	4	Neutral	−1.392
rs727503106	R97K	tolerated	0.29	effect	53	Neutral	−2.130
**rs727503108**	**G60V**	**intolerant**	**0**	**effect**	**77**	**Deleterious**	**−8.727**
**rs727503110**	**Q22R**	**intolerant**	**0**	**effect**	**20**	**Deleterious**	**−3.324**
**rs727503110**	**Q22L**	**intolerant**	**0**	**effect**	**49**	**Deleterious**	**−5.834**
**rs727504662**	**M72L**	**intolerant**	**0.01**	**effect**	**46**	**Deleterious**	**−2.549**
rs730880470	T50S	tolerated	0.51	neutral	-44	Deleterious	−3.144
**rs730880471**	**D119N**	**intolerant**	**0**	**effect**	**83**	**Deleterious**	**−4.566**
**rs730880472**	**L23R**	**intolerant**	**0**	**effect**	**77**	**Deleterious**	**−4.925**
rs730880473	A130V	intolerant	0.03	effect	17	Neutral	−2.446
**rs770248150**	**K117N**	**intolerant**	**0**	**effect**	**86**	**Deleterious**	**−4.558**
**rs794727277**	**N26Y**	**intolerant**	**0.01**	**effect**	**51**	**Deleterious**	**−5.455**
**rs794727720**	**Y157C**	**intolerant**	**0.02**	**effect**	**3**	**Deleterious**	**−6.787**
rs1057517885	I171T	tolerated	0.59	neutral	-20	Neutral	0.121
**rs1057519725**	**A146V**	**intolerant**	**0**	**effect**	**53**	**Deleterious**	**−3.625**
**rs1135401776**	**K147R**	**intolerant**	**0.01**	**effect**	**18**	**Deleterious**	**−2.718**
**rs138669124**	**F141L**	**intolerant**	**0.01**	**effect**	**65**	**Deleterious**	**−4.534**
rs368557003	Q165R	tolerated	0.85	neutral	-56	Neutral	−0.013
**rs373500216**	**A134G**	**intolerant**	**0.04**	**effect**	**53**	**Deleterious**	**−3.378**
rs374681135	P178S	tolerated	0.77	neutral	-29	Neutral	−0.199
rs529925358	I183V	tolerated	0.47	effect	5	Neutral	0.071
rs539423712	V160A	tolerated	0.05	effect	28	Deleterious	−3.533
rs542902732	M1I	intolerant	0.01	neutral	-13	Neutral	−2.480
rs575569675	T124S	tolerated	0.65	neutral	-41	Neutral	−0.801
rs746609817	K128R	tolerated	0.27	effect	11	Neutral	−0.496
rs749177256	T158I	tolerated	0.14	effect	5	Deleterious	−4.132
**rs754870563**	**G138E**	**intolerant**	**0.02**	**effect**	**41**	**Deleterious**	**−5.915**
rs755177746	A155G	tolerated	0.08	effect	53	Deleterious	−3.310
**rs755877953**	**V160M**	**intolerant**	**0**	**effect**	**20**	**Deleterious**	**−2.648**
rs755967833	I188V	intolerant	0.5	effect	26	Neutral	−0.257
**rs756890312**	**G77A**	**intolerant**	**0.01**	**effect**	**58**	**Deleterious**	**−5.942**
rs757674707	V160I	intolerant	0.04	effect	19	Neutral	−0.885
rs757816355	S136N	tolerated	0.3	neutral	-26	Neutral	−1.842
rs766231905	I171M	tolerated	0.12	neutral	-26	Neutral	0.484
rs770020203	T74A	tolerated	0.13	effect	21	Deleterious	−4.434
rs771629239	E174K	tolerated	0.58	neutral	-47	Neutral	−0.392
**rs772985440**	**S172C**	**intolerant**	**0.02**	**effect**	**16**	**Deleterious**	**−2.706**
rs775836436	V112I	tolerated	0.47	neutral	-14	Neutral	−0.580
rs778702415	G138R	tolerated	0.07	effect	20	Deleterious	−5.963
rs779951033	I187V	tolerated	1	neutral	-69	Neutral	0.083
**rs780974222**	**G75A**	**intolerant**	**0.02**	**effect**	**34**	**Deleterious**	**−5.938**
rs781634879	T127R	tolerated	0.63	effect	3	Deleterious	−2.928
**rs868857258**	**L79P**	**intolerant**	**0**	**effect**	**76**	**Deleterious**	**−6.881**
**rs904755552**	**I46M**	**intolerant**	**0**	**effect**	**20**	**Deleterious**	**−2.794**
rs953088090	K88E	tolerated	0.24	effect	47	Deleterious	−2.537
**rs989151052**	**D154G**	**intolerant**	**0**	**effect**	**57**	**Deleterious**	**−4.537**
rs1024789250	K182E	tolerated	0.23	effect	35	Neutral	−1.118
rs1191739287	K170E	tolerated	0.83	effect	2	Neutral	−0.754
**rs1199162369**	**R68C**	**intolerant**	**0**	**effect**	**68**	**Deleterious**	**−7.900**
rs1265970615	T158P	tolerated	0.23	effect	53	Deleterious	−3.632
rs1296330213	L6I	tolerated	0.09	neutral	-53	Neutral	−1.566
rs1296330213	L6V	tolerated	0.06	effect	3	Neutral	−2.337
**rs1307793966**	**R164G**	**intolerant**	**0.01**	**effect**	**72**	**Deleterious**	**−4.743**
rs1308177469	M189I	tolerated	0.24	effect	11	Neutral	−0.817
rs1309399018	H95N	tolerated	0.5	effect	2	Neutral	0.028
**rs1340281106**	**N86H**	**intolerant**	**0.04**	**effect**	**64**	**Deleterious**	**−3.195**
rs1344202459	I142T	tolerated	0.1	effect	26	Deleterious	−3.283
**rs1363431968**	**D126H**	**intolerant**	**0.03**	**effect**	**59**	**Deleterious**	**−3.361**
rs1407509439	T50I	tolerated	0.15	neutral	-20	Deleterious	−3.410
rs1434157586	R123W	tolerated	0.08	effect	52	Deleterious	−6.229
**rs1437657227**	**D92H**	**intolerant**	**0**	**effect**	**37**	**Deleterious**	**−4.092**
**rs1463850736**	**A130T**	**intolerant**	**0**	**effect**	**32**	**Deleterious**	**−2.556**
**rs1463850736**	**A130P**	**intolerant**	**0.01**	**effect**	**51**	**Deleterious**	**−3.248**
rs1463850736	A130S	intolerant	0.01	effect	24	Neutral	−1.591
rs1470495974	I163V	tolerated	0.24	neutral	-22	Neutral	−0.817

**Table 2:** Results of the evolutionary conservation analyses using the ConSurf server. SNPs indicated in bold are predicted to be highly conserved and are selected for further evaluation.

rs ID	Variant	Conservation Score		
		SWISS-PROT	UniProt	UniRef90
**rs17851045**	**Q61H**	**9**	**8**	**9**
**rs104886029**	**A59V**	**9**	**9**	**9**
**rs104894359**	**G60S**	**9**	**9**	**9**
**rs104894359**	**G60R**	**9**	**9**	**9**
**rs104894361**	**K5N**	**8**	**8**	**8**
rs104894362	F156L	9	9	9
**rs104894364**	**T58I**	**9**	**9**	**9**
rs104894366	P34R	6	6	5
rs104894367	V152G	8	9	8
rs112445441	G13D	6	6	6
**rs121913236**	**Q22K**	**8**	**7**	**7**
**rs121913238**	**Q61K**	**9**	**8**	**9**
**rs121913239**	**Q61E**	**9**	**8**	**9**
**rs121913240**	**Q61P**	**9**	**8**	**9**
**rs121913241**	**Q61R**	**9**	**8**	**9**
**rs121913242**	**Q61L**	**9**	**8**	**9**
**rs121913527**	**A146P**	**9**	**9**	**9**
**rs121913528**	**A59T**	**9**	**9**	**9**
**rs121913528**	**A59S**	**9**	**9**	**9**
**rs121913529**	**G12D**	**7**	**8**	**8**
**rs121913531**	**G12A**	**7**	**8**	**8**
**rs121913534**	**G12V**	**7**	**8**	**8**
**rs121913530**	**G12S**	**7**	**8**	**8**
**rs121913532**	**G12R**	**7**	**8**	**8**
**rs121913533**	**G12C**	**7**	**8**	**8**
rs121913535	G13C	6	6	6
rs121913538	L19F	6	7	7
**rs193929331**	**K5E**	**8**	**8**	**8**
**rs202247812**	**N116S**	**9**	**9**	**9**
rs387907205	Y71H	8	7	7
rs387907205	Y71D	8	7	7
**rs387907206**	**K147E**	**8**	**7**	**7**
rs397517041	V152F	8	9	8
rs397517042	F156I	9	9	9
rs397517042	F156V	9	9	9
**rs727503108**	**G60V**	**9**	**8**	**9**
**rs727503110**	**Q22R**	**8**	**7**	**7**
**rs727503110**	**Q22L**	**8**	**7**	**7**
rs727504662	M72L	8	7	7
**rs730880471**	**D119N**	**9**	**9**	**9**
rs730880472	L23R	7	7	7
**rs770248150**	**K117N**	**9**	**9**	**9**
rs794727277	N26Y	7	4	5
rs794727720	Y157C	1	1	1
**rs1057519725**	**A146V**	**9**	**9**	**9**
**rs1135401776**	**K147R**	**8**	**7**	**7**
rs138669124	F141L	7	6	7
rs373500216	A134G	8	8	8
rs754870563	G138E	1	1	1
rs755877953	V160M	6	7	7
rs756890312	G77A	7	7	7
rs372793780	R164Q	4	6	5
rs772985440	S172C	1	1	1
rs780974222	G75A	7	7	7
rs868857258	L79P	6	5	5
rs904755552	I46M	5	5	5
rs989151052	D154G	2	1	3
**rs1199162369**	**R68C**	**8**	**8**	**7**
rs1307793966	R164G	4	6	5
rs1340281106	N86H	5	5	5
rs1363431968	D126H	3	3	4
rs1437657227	D92H	6	5	5
rs1463850736	A130T	5	6	6
rs1463850736	A130P	5	6	6

**Table 3:** Results of the analyses using the I-Mutant2.0 and Mupro. SNPs indicated in bold are predicted to decrease the stability of the protein structure and are selected for further evaluation.

rs ID	Variant	I-Mutant2.0		Mupro	
		DDG	Stability	DDG	Stability
**rs17851045**	**Q61H**	**−0.96**	**Decrease**	**−0.48**	**Decrease**
**rs104886029**	**A59V**	**−0.06**	**Decrease**	**−0.35**	**Decrease**
**rs104894359**	**G60S**	**−1.39**	**Decrease**	**−0.78**	**Decrease**
**rs104894359**	**G60R**	**−1.36**	**Decrease**	**−0.63**	**Decrease**
**rs104894361**	**K5N**	**−0.25**	**Decrease**	**−0.70**	**Decrease**
rs104894364	T58I	0.28	Increase	−0.003	Decrease
**rs121913236**	**Q22K**	**−0.55**	**Decrease**	**−1.27**	**Decrease**
**rs121913238**	**Q61K**	**−0.16**	**Decrease**	**−0.56**	**Decrease**
rs121913239	Q61E	0.22	Increase	−0.13	Decrease
**rs121913240**	**Q61P**	**−1.35**	**Decrease**	**−0.69**	**Decrease**
**rs121913241**	**Q61R**	**−0.55**	**Decrease**	**−0.18**	**Decrease**
rs121913242	Q61L	0.47	Increase	0.54	Increase
**rs121913527**	**A146P**	**−1.58**	**Decrease**	**−1.32**	**Decrease**
**rs121913528**	**A59T**	**−1.36**	**Decrease**	**−1.34**	**Decrease**
**rs121913528**	**A59S**	**−0.82**	**Decrease**	**−1.03**	**Decrease**
**rs121913529**	**G12D**	**−0.83**	**Decrease**	**−0.75**	**Decrease**
**rs121913531**	**G12A**	**−0.53**	**Decrease**	**−1.19**	**Decrease**
**rs121913534**	**G12V**	**−0.36**	**Decrease**	**−0.66**	**Decrease**
**rs121913530**	**G12S**	**−1.49**	**Decrease**	**−1.08**	**Decrease**
**rs121913532**	**G12R**	**−1.47**	**Decrease**	**−0.93**	**Decrease**
**rs121913533**	**G12C**	**−1.34**	**Decrease**	**−0.58**	**Decrease**
**rs193929331**	**K5E**	**−0.23**	**Decrease**	**−0.43**	**Decrease**
**rs202247812**	**N116S**	**−0.81**	**Decrease**	**−1.84**	**Decrease**
**rs387907206**	**K147E**	**−0.73**	**Decrease**	**−0.38**	**Decrease**
**rs727503108**	**G60V**	**−1.22**	**Decrease**	**−0.38**	**Decrease**
**rs727503110**	**Q22R**	**−1.31**	**Decrease**	**−0.84**	**Decrease**
**rs727503110**	**Q22L**	**−0.08**	**Decrease**	**−0.10**	**Decrease**
**rs730880471**	**D119N**	**−1.43**	**Decrease**	**−0.78**	**Decrease**
**rs770248150**	**K117N**	**−0.68**	**Decrease**	**−0.17**	**Decrease**
**rs1057519725**	**A146V**	**−0.34**	**Decrease**	**−0.97**	**Decrease**
**rs1135401776**	**K147R**	**−0.63**	**Decrease**	**−0.41**	**Decrease**
rs1199162369	R68C	0.33	Increase	−1.33	Decrease

**Table 4:** Time averaged structural properties calculated for wild-type (WT), Q22K, Q61P, and Q61R.

	WT	Q22K	Q61P	Q61R
**Backbone rmsd (nm)**	0.1853(0.0187)	0.2108(0.0771)	0.2504(0.0652)	0.2240(0.0741)
**Cα-rmsd (nm)**	0.1933(0.0195)	0.2173(0.0766)	0.2562(0.0654)	0.2294(0.0743)
**Cα-rmsf (nm)**	0.0939(0.0666)	0.1251(0.1068)	0.1183(0.0985)	0.1289(0.0845)
**Rg-Cα (nm)**	1.4960(0.0086)	1.5072(0.0169)	1.5130(0.0181)	1.5128(0.0148)
**Rg-protein (nm)**	1.5495(0.0087)	1.5629(0.0193)	1.5686(0.0209)	1.5658(0.0145)
**SASA (nm^2^)**	93.008(1.9027)	94.806(3.0942)	96.109(3.2164)	96.159(2.7846)

RMSD: root mean square deviation; RMSF: root mean square fluctuation; Rg: radius of gyration; SASA: solvent accessible surface area.

**Table 5:** Trajectory-averaged percentages of secondary structures of the simulated WT and MTs system.

Sample	%				
	α-Helices	β-Sheets	Coils	Bend	Turn
Wild	35.61	23.53	20.04	10.07	9.14
Q22K	34.94	24.63	19.60	9.26	9.89
Q61P	34.65	23.19	19.12	11.24	10.00
Q61R	35.27	23.23	20.41	10.12	9.37

## Supplementary Materials

The following are available online. Figure S1: Interaction plot of wild-type and the mutated residues with the neighborhood; Figure S2: The Number of hydrogen bonds for WT and MTs with respect to simulation time.
