# Supplementary material for: Computational Screening and Analysis of Lung Cancer Related Non-Synonymous Single Nucleotide Polymorphisms on the Human Kirsten Rat Sarcoma Gene
Source: Molecules. 2019 May 21;24(10):1951. doi: 10.3390/molecules24101951 (PMC6572712; doi:10.3390/molecules24101951)
Supplement: Supplementary file 1 [file molecules-24-01951-s001.pdf]

molecules

Supplementary

# Computational Screening and Analysis of Lung Cancer Related Non-Synonymous Single Nucleotide Polymorphisms on Human KRAS Gene

**Qiankun Wang, Aamir Mehmood, Heng Wang, Qin Xu, Yi Xiong* and Dong-Qing Wei***

State Key Laboratory of Microbial Metabolism, School of Life Sciences and Biotechnology, Shanghai Jiao Tong University, Shanghai 200240, China; wangqiankun@sjtu.edu.cn (Q.W.); aamirmehmood@sjtu.edu.cn, (A.M.); wangheng0802@sjtu.edu.cn, (H.W.); xuqin523@sjtu.edu.cn (Q.X.)

* Correspondence: xiongyi@sjtu.edu.cn (Y.X.); dqwei@sjtu.edu.cn (D.-Q.W.); Tel.: +86-21-3420-4573 (Y.X. & D.-Q.W.)

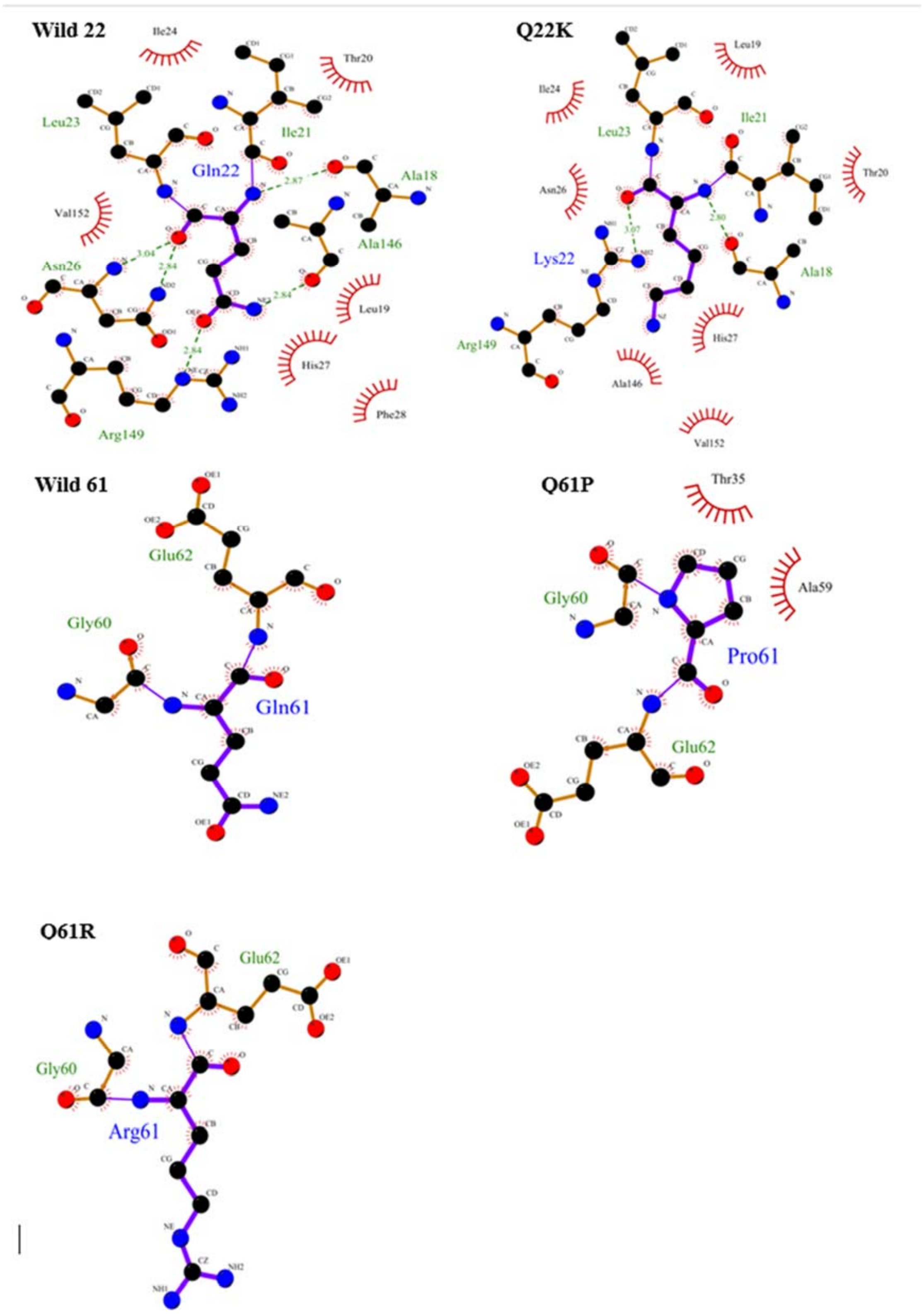


**Figure S1.** Interaction plot of wild-type and the mutated residues with the neighborhood

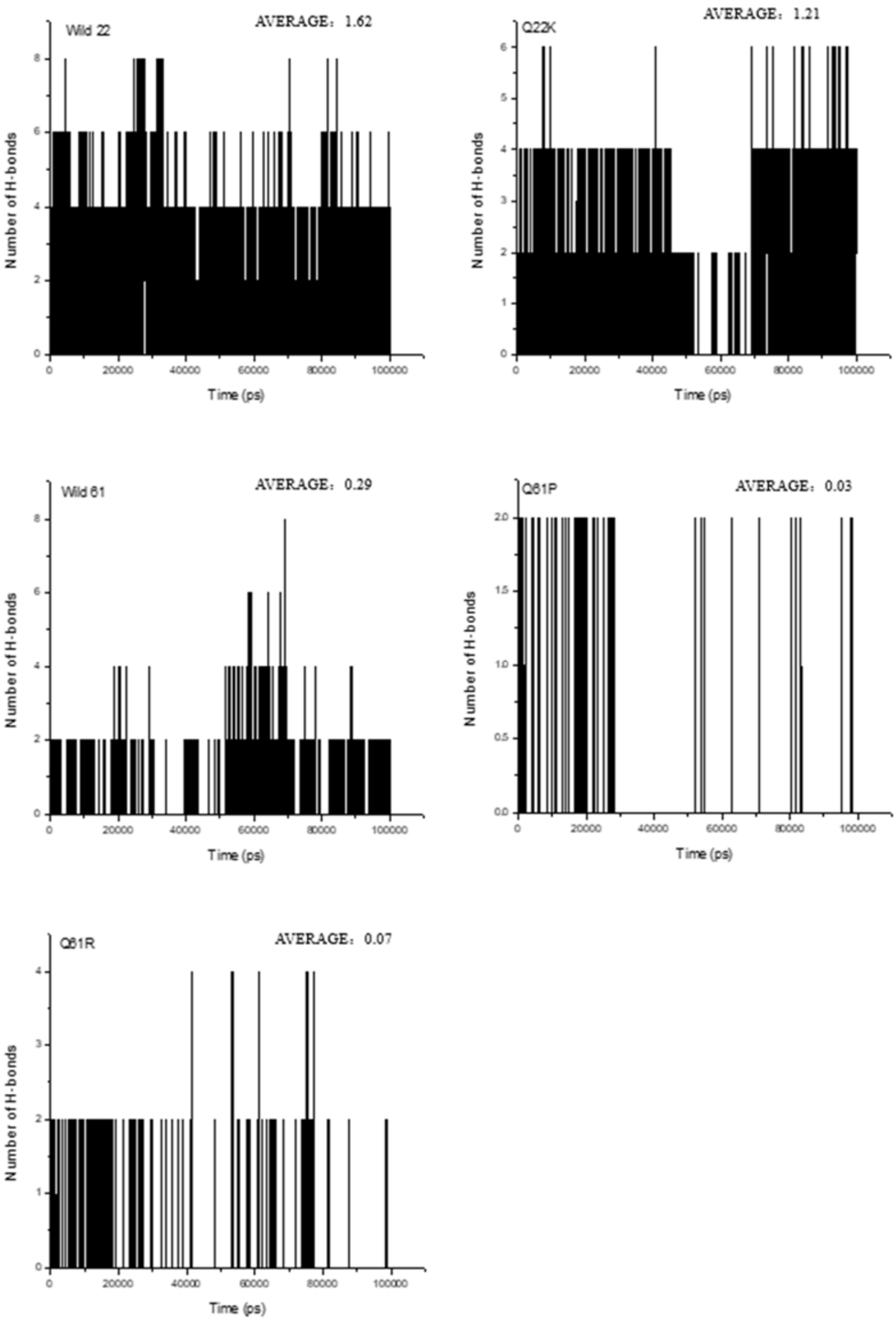


**Figure S2.** The Number of hydrogen bonds for WT and MTs with respect to simulation time
